# Recurrence or *de novo*? Intradermal Basal Cell Carcinoma of the Scrotum: A Report of Two Cases

**DOI:** 10.3390/dermatopathology10020019

**Published:** 2023-03-28

**Authors:** Kaori Yamazaki, Maho Maejima, Hidehisa Saeki, Shin-Ichi Osada

**Affiliations:** 1 Department of Dermatology, Nippon Medical School Tama Nagayama Hospital, Tokyo 206-8512, Japan; 2 Department of Dermatology, Nippon Medical School, Tokyo 113-8603, Japan

**Keywords:** basal cell carcinoma, scrotum, recurrence, stem cells, nodular, micronodular

## Abstract

Basal cell carcinoma (BCC) is the most common cutaneous malignancy, usually occurring in sun-exposed areas. Although BCC in the scrotal region is uncommon, it carries a higher risk of metastasis than BCC at other sites. Here, we report two cases of BCC that developed in the scrotal region: Case 1 presented as a superficial nodule and Case 2 as a subcutaneous nodule. Histopathologically, both tumors lacked continuity with the surface epidermis and formed an intradermal nodule. In Case 1, BCC occurred for the first time and presumably developed *de novo*. Case 2 underwent excision of a scrotal BCC 5 years previously, and the histopathological diagnosis at that time was nodular BCC. However, when the original specimen was re-examined, it was determined that, although the tumor had been completely resected, part of the lesion had moved away from the nodular area to represent a micronodular phenotype, an aggressive BCC subtype. We hypothesized that partial evolution from a nodular to a micronodular phenotype may have contributed to the recurrence of BCC in Case 2.

## 1. Introduction

Basal cell carcinoma (BCC) is the most prevalent cutaneous malignancy worldwide [1,2]. As chronic exposure to ultraviolet light is a major risk factor for its development, most cases occur in chronically sun-exposed areas, such as the head and neck, in the elderly [1,2], whereas it is rarely observed in non-sun-exposed areas, such as the genital region [3]. A classic review reported that less than 0.5% of BCCs were located in the genital area [4], and a recent population-based analysis clarified that scrotal BCCs accounted for 74.5% of genital BCCs [5].

The most common clinical presentations of scrotal BCC—plaques, nodules, and ulcers—are non-specific; hence, they tend to be misdiagnosed as chronic eczema or other unrelated skin conditions. The differential diagnoses of benign tumors potentially arising in the scrotal region include verruca vulgaris, condyloma acuminatum, bowenoid papulosis, pyogenic granuloma, angiokeratoma scroti, and verruciform xanthoma. Besides BCC, the most common scrotal malignancies are squamous cell carcinoma, extramammary Paget’s disease, and Bowen’s disease [6]. The median time from detecting the scrotal lesion to confirming the diagnosis of BCC is approximately 12 months, with a range from 3 months to many years [3]. Owing to the long time taken to diagnose the lesions, scrotal BCCs are characterized by relatively large tumor sizes, ranging from 1.5 to 5 cm in diameter [7].

BCC has several histopathological subtypes. In a review of 1039 consecutive BCC cases, the most common subtypes were mixed (38.5%), nodular (21.0%), superficial (17.4%), micronodular (14.5%), infiltrative (7.4%), and morphoeic (1.1%) [8]. Dermoscopic analysis is a non-invasive skin examination procedure that is useful for the diagnosis of BCC, with a sensitivity of 91.2% and a specificity of 95% [9]. The dermoscopic features indicative of BCC are arborizing vessels (the most prevalent feature), short fine telangiectasia, shiny white structures, large blue-gray ovoid nests, multiple blue-gray globules/dots, spoke wheel area, leaf-like areas, or concentric structures [10,11]. These features vary in prevalence and distribution among BCC subtypes. The overall dermoscopic appearance of BCC is represented by a mixture of the abovementioned features. According to the risk of recurrence, BCC is classified into low-risk subtypes (nodular, superficial, pigmented, infundibulocystic, and fibroepithelial) and high-risk subtypes (infiltrating, micronodular, sclerosing/morphoeic, basosquamous, and BCC with sarcomatoid differentiation) [12]. The majority of reported scrotal BCCs are nodular, with only a minority of them being micronodular, infiltrating, and sclerosing/morphoeic [3,7,13,14].

The pathogenesis of scrotal BCC remains unknown. Although ultraviolet light, radiotherapy, chronic exposure to coal tar and arsenic, fair complexion, and genetic predisposition are known risk factors for the development of BCC [2], reported cases of scrotal BCC do not show a direct relationship with these risk factors [3]. In addition, a large study failed to detect the human papillomavirus in skin biopsies from scrotal BCC [3]. Therefore, scrotal BCC may result from non-specific factors such as poor hygiene, local chronic skin irritation, and reduced immune surveillance due to increasing age [3,7].

Metastasis of BCC is rare, with metastasis rates ranging from 0.0028% to 0.55% [7]. However, BCC in the scrotal region is considered high-risk, with metastases occurring more frequently and earlier than in other parts of the body [3,4,5,6,15]. Metastases, most commonly to the local lymph nodes and lungs, have been reported in 13–20% of patients with scrotal BCC 2–3 years after diagnosis, compared with approximately 0.003–0.5% after 9–11 years for BCC at other sites [3]. This high incidence of metastases from scrotal BCC suggests that BCC located on the scrotum is more aggressive than BCC located elsewhere. This may be because the thin scrotal skin lacks subcutaneous fat and is well-vascularized [3]. Involvement of the dartos muscle may be a poor prognostic indicator of the final pathology [7].

Here, we report two cases of scrotal BCC. Although they showed different clinical presentations: a superficial nodule in Case 1 and a subcutaneous nodule in Case 2; histopathologically, neither tumor had any connection to the surface epidermis, appearing as an intradermal nodular structure. The BCC in Case 1 was the first occurrence in that patient and presumably developed *de novo*, whereas the patient in Case 2 had undergone excision of the scrotal BCC 5 years previously. Although BCC recurrence often occurs as a result of incomplete excision [16], the subcutaneous nodule appeared further away from the excision scar, and the tumor had been completely excised at the initial surgery. Therefore, several questions remain to be answered before recurrence is confirmed in Case 2. Here, we discuss the reasons for both scrotal BCCs presenting as intradermal nodules.

## 2. Case Presentation

### 2.1. Case 1

**Figure 1:**
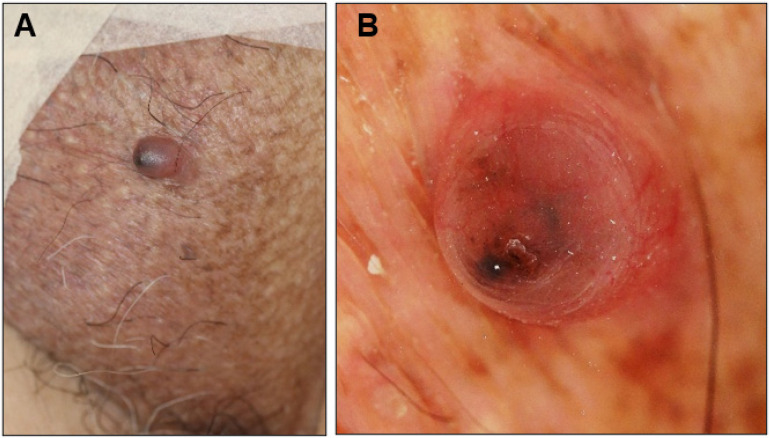
Clinical presentation (**A**) and dermoscopic view (**B**) of the scrotal nodule in Case 1.

An 81-year-old man presented with a 1-year history of an asymptomatic small protrusion on the scrotum. He had a history of hypertension, diabetes mellitus, and Parkinson’s disease. On examination, a 7 × 6 mm reddish slightly firm smooth-surfaced nodule with a black tip was found on the left side of the scrotum (Figure 1A). No lymphadenopathy was observed. The differential diagnosis included BCC, angiokeratoma scroti, and pyogenic granuloma. Dermoscopic analysis showed large blue-gray ovoid nests and short fine telangiectasias (Figure 1B), which are characteristic dermoscopic features of BCC. The nodule was excised with a 3 mm margin.

**Figure 2:**
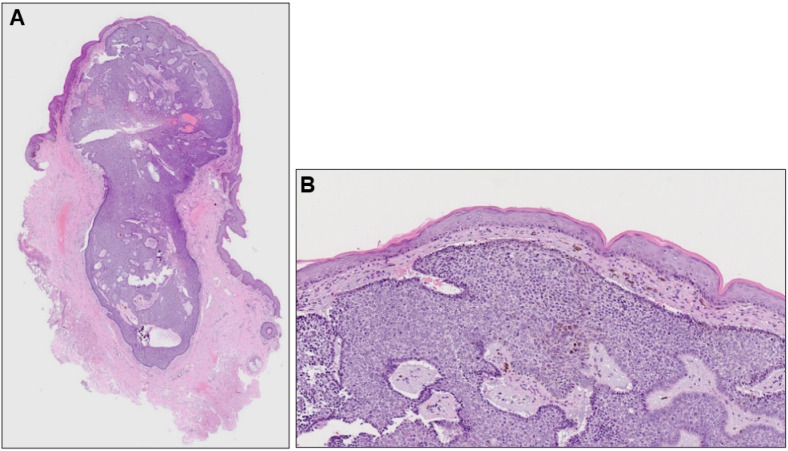
(**A**) Histopathological image of the nodule (hematoxylin-eosin stain, ×40) and its higher magnification (**B**, ×100).

Histopathological examination revealed a well-demarcated, partially hemorrhagic nodule of basophilic tumor cells in the dermis (Figure 2A). At higher magnification, nuclei were arranged in a palisade pattern at the periphery of the tumor nests and cleft formation was observed in the surrounding stroma (Figure 2B). However, the tumor nests lacked continuity with the epidermis. Based on these clinical, dermoscopic, and histopathological findings, a diagnosis of nodular BCC was made. Six months after surgery, there was no recurrence.

### 2.2. Case 2

**Figure 3:**
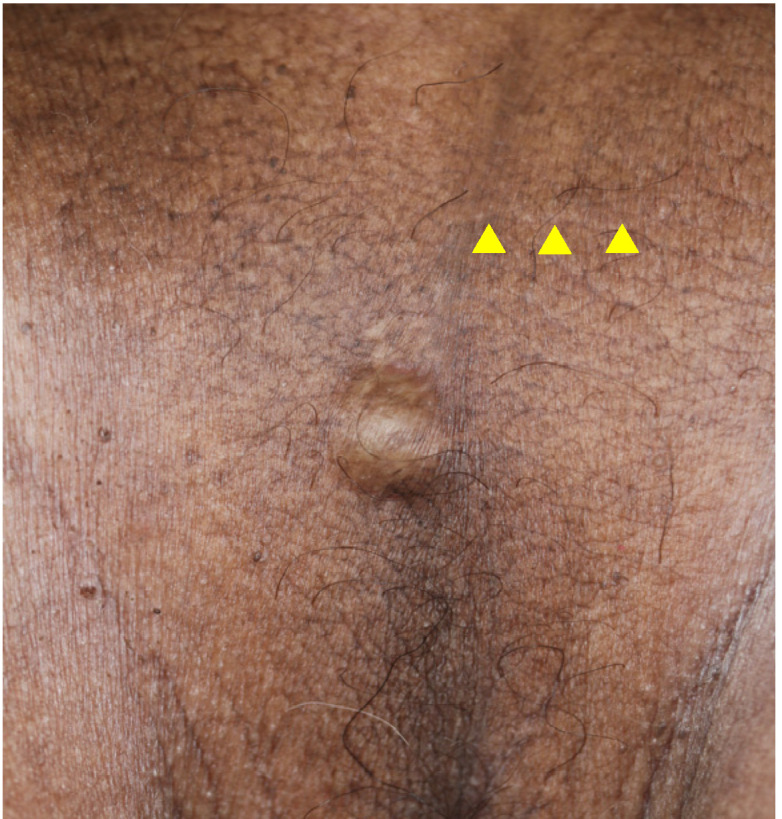
Clinical picture of Case 2 at the time of the consultation. The yellow triangles indicate the postoperative scar.

A 73-year-old man with a subcutaneous nodule in the scrotal region was referred to our department. He had undergone excision of a scrotal tumor 5 years previously at a nearby non-dermatology clinic. The excised tumor was histopathologically diagnosed as nodular BCC. Upon examination at our department, a previous postoperative scar was visible on the scrotum and a 15 × 15 mm slightly firm dome-shaped nodule was palpable near the scrotal suture line below the scar (Figure 3). The differential diagnoses included follicular cyst, lipoma, leiomyoma, recurrence of BCC, and metastatic skin tumor. The inguinal lymph nodes were not palpable and skin ultrasonography revealed no enlarged lymph nodes. The tumor was excised with a surrounding skin margin of 5.0 mm.

**Figure 4:**
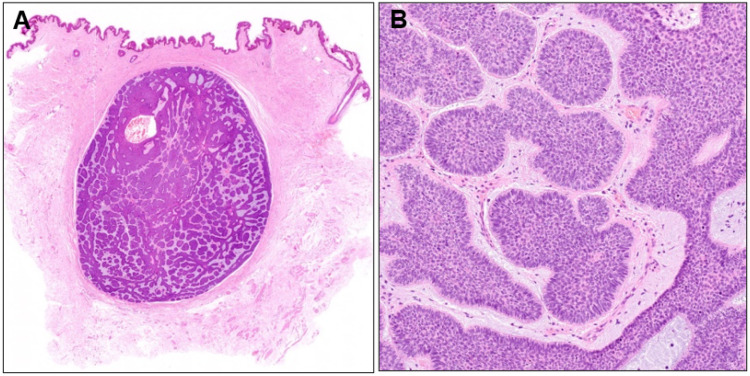
(**A**) Histopathological image of the subcutaneous nodule (×40) and its higher magnification (**B**, ×100).

Histopathological examination revealed a well-demarcated nodule of basophilic tumor cells in the dermis surrounded by fibrous tissue (Figure 4A). A diagnosis of nodular BCC was made because the nuclei at the periphery of the tumor nests were arranged in a palisade pattern, and mucin deposition and fissure formation were observed around the tumor nests (Figure 4B). Although the entire tumor had a nodular structure, small tumor nests were surrounded by a thin stromal rim in some areas. Two years have passed since the second surgery, without any evidence of recurrence.

**Figure 5:**
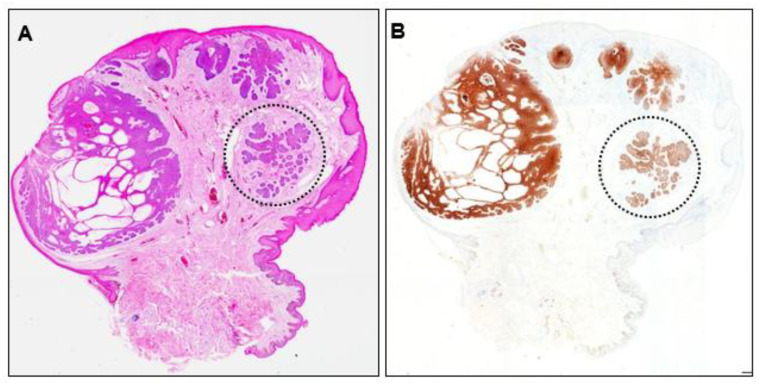
(**A**) Histopathological image of the initially resected specimen (×40) and (**B**) Ber-EP4 immunohistochemistry image (×40). Dotted circles indicate tumor nests with a micronodular phenotype.

Recurrence of the initial scrotal BCC was suspected and the originally resected specimen was reviewed. The lesion showed partial ulceration of the epidermis and internal cystic structures, and the overall structure was nodular BCC (Figure 5A). No residual tumor cells were observed at the excision margins. No residual Ber-EP4-positive cells were found, even in deeper sections (Figure 5B), indicating complete resection. However, closer examination revealed an area within the lesion that lacked continuity with the epidermis. This area consisted of small tumor nests surrounded by fibrous tissue mimicking a micronodular phenotype (Figure 5, dotted circle).

## 3. Discussion

We have reported two cases of scrotal BCC presenting as a superficial nodule in Case 1 and as a subcutaneous nodule in Case 2. Both patients had no history of sexually transmitted diseases, ionizing radiation to the genital region, immunosuppressive therapy, or history of exposure to arsenic or coal tar derivatives. Although more than 90% of BCCs have a connection between tumor cell formation and the surface epidermis [17], both tumors lacked continuity with the epidermis and formed an intradermal nodule. The blurred dermoscopic image in Case 1 is thought to reflect the presence of the epidermis overlying the tumor nests (Figure 1B). The reported scrotal BCCs formed erythema, nodules, papules, plaques, or ulcers on the surface of the scrotum and, to the best of our knowledge, no scrotal BCCs with intradermal nodule formation have been reported. However, we assume that the mechanisms for intradermal nodule formation are different in the two cases.

The absence of continuous tumor formation from the epidermis in Case 1 suggests that this patient did not receive any pathogenic insults to the scrotal surface. He had no history of scrotal trauma, radiotherapy, chemotherapy, or exposure to chemicals. Thus, the intradermal tumor cell formation, in this case, may reflect the process in which a tumor arising within the dermis migrated towards the epidermis.

The mechanism by which the tumor developed in the dermis is not known, but it is possible that it originated from what are called “stem cells”. In 1948, Lever expressed the idea that BCC is derived from primary epithelial germ cells [18]. Originally, it was thought that the primary epithelial germ cells that give rise to BCC were embryonic cells that remained dormant until the onset of neoplasia. Subsequently, Pinkus suggested that BCCs occurring later in life arise not from dormant embryonic primary epithelial germ cells, but from pluripotent cells that form continuously during life [19]. These seminal ideas regarding the origin of BCCs have evolved into the current stem cell theory.

Loss of heterozygosity of patched or activating mutations in smoothened gene (SmoM2) leading to constitutive activation of the hedgehog (HH) pathway is found in most sporadic or inherited forms of BCC [2]. Because mouse models that recapitulate these genetic aberrations observed in human BCCs have yielded contradictory results, the precise cellular origin of the stem cells of BCC remains controversial [20,21,22]. SmoM2-induced BCC-like tumors appear to arise from stem cells in the interfollicular epidermis (IFE) but not from the hair follicle bulge [20], whereas tumors driven by the loss of patched1 have been reported to originate from the bulge and secondary hair germ but not the IFE [21]. BCCs also arise from stem cells within touch dome epithelia, an innervated subset of IFE cells [22]. Consistent with the idea that stem cells for BCC have hair follicle stem cell origin, BCC expresses the epithelial cell adhesion molecule (Ep-CAM) similar to the early stages of the embryonic human hair follicle, the secondary hair germ, and the outer root sheath of the vellus hair follicle [23].

Because Case 2 had a history of scrotal BCC excision, it would be rational to assume that this was a recurrence. Previous reports have indicated that incomplete excision is the most common cause of recurrence of BCC [16]. However, in this case, some questions remain to be answered before recurrence can be assumed. First, the subcutaneous tumor originated from a previous surgical scar. Second, examination of the initial specimen showed that the tumor margins were clear of malignant cells both laterally and inferiorly; thus, incomplete resection was unlikely to be the cause. Third, a review of the initial pathology specimen identified areas with a partially aggressive phenotype.

BCC has several histopathological low- and high-risk subtypes according to their risk of recurrence: the nodular, superficial, pigmented, infundibulocystic, and fibroepithelial subtypes are considered low-risk (non-aggressive), whereas the micronodular, sclerosing/morphoeic, basosquamous, and infiltrating subtypes are considered high-risk (aggressive) [12]. In this case, review of the original resected specimen revealed that the overall architecture was nodular, and most of the tumor was continuous with the epidermis. However, some areas lacked continuity with the epidermis and consisted of small tumor nests surrounded by fibrous tissue (Figure 5), suggesting that this area may have acquired the micronodular phenotype. Because the element that accounts for more than 50% of the total tumor should be given priority in determining the subtype, the diagnosis of nodular BCC is reasonable [17].

Micronodular BCC refers to lesions with multiple small aggregates of basaloid cells within the dermis, often with no appreciable connection to the overlying epidermis. Compared with nodular BCC lesions, peripheral palisading and retraction artifacts are subtle [1]. Clinically, micronodular BCCs are often difficult to distinguish from superficial and nodular BCCs because they can present as erythematous macules or thin plaques. Dermoscopically, milky red structureless areas (53.8%), arborizing vessels and short fine telangiectasias (53.8% and 50%, respectively), ulceration (46.2%), and blue structure (57.7%) were commonly observed [24]. Owing to their multifocal nature, these lesions often have subclinical extension and, consequently, higher recurrence rates. Occurring in an estimated 15% of BCCs, micronodular changes are often observed together with other histopathologic patterns [1]. Typically, micronodular differentiation is observed towards the base of the tumor [17]. This was also true for Case 2. Most reported scrotal BCCs are of the nodular type, with only a minority being of micronodular types [4,9]. However, because the nodular type can have an element of the micronodular type, as in this case, careful histopathological examination is necessary to predict and prevent recurrence and metastasis.

The mechanisms by which aggressive BCCs develop are beginning to be fully elucidated. It was recently reported that transcriptional reprogramming occurs at the leading edge of the tumor–stroma interface in infiltrative BCC cases, thereby giving rise to tumor cells with a collective migratory phenotype and cancer-associated fibroblasts with extracellular matrix remodeling properties [25]. Moreover, the activin A signaling pathway regulates transcriptional tumor–stroma interactions and spatial organization. Therefore, it is plausible that, in the present case, transcriptional reprogramming occurred in some areas of the nodular BCC, acquiring a more aggressive micronodular phenotype. As a result, the aggressive tumor cell population that migrated into the dermis escaped resection during the initial surgery and later formed an intradermal nodule.

Alternatively, we emphasize that the subcutaneous nodule of BCC in Case 2 arose near the scrotal suture. The scrotum is formed by fusion of the scrotal suture at approximately 12 weeks of gestation. Therefore, as in Case 1, it is conceivable that stem cells that migrated aberrantly into the dermis during scrotal suture formation may have become BCC as a result of chronic inflammation caused by an unsanitary environment, decreased immune surveillance due to aging, or other stimuli.

The risk of focal recurrence may be related to perineural invasion. Although perineural invasion was not found in our two cases, we believe that scrotal BCCs should continue to be followed up carefully as they are at higher risk of recurrence and metastasis than BCCs arising in other sites. The low incidence of scrotal BCC makes it difficult to validate the mechanisms of scrotal BCC development that we have discussed here; however, we await further accumulation and analysis of cases.

## 4. Conclusions

Herein, we report two cases of scrotal BCC with histopathological intradermal nodules. Case 1 suggested that the scrotal BCC developed *de novo* and was derived from tumor stem cells. In Case 2, we observed a partial evolution from the nodular to the micronodular subtype within the tumor, which may explain the recurrence of the previous scrotal BCC. Careful histopathological examination is important for predicting the prognosis of scrotal BCC.

## Data Availability

The data presented in this study are available on request from the corresponding author. The data are not publicly available due to privacy reasons.
